# Arbitrarily Accessible 3D Microfluidic Device for Combinatorial High-Throughput Drug Screening

**DOI:** 10.3390/s16101616

**Published:** 2016-09-29

**Authors:** Zhuofa Chen, Weizhi Li, Gihoon Choi, Xiaonan Yang, Jun Miao, Liwang Cui, Weihua Guan

**Affiliations:** 1 Department of Electrical Engineering, Pennsylvania State University, University Park, PA 16802, USA; zjc5117@psu.edu (Z.C.); gmc5400@psu.edu (G.C.); xuy113@psu.edu (X.Y.); 2 Department of Entomology, Pennsylvania State University, University Park, PA 16802, USA; wxl23@psu.edu (W.L.); jum23@psu.edu (J.M.); luc2@psu.edu (L.C.); 3 Department of Biomedical Engineering, Pennsylvania State University, University Park, PA 16802, USA

**Keywords:** microfluidic, high-throughput, combinatorial, multiplex, drug screening

## Abstract

Microfluidics-based drug-screening systems have enabled efficient and high-throughput drug screening, but their routine uses in ordinary labs are limited due to the complexity involved in device fabrication and system setup. In this work, we report an easy-to-use and low-cost arbitrarily accessible 3D microfluidic device that can be easily adopted by various labs to perform combinatorial assays for high-throughput drug screening. The device is capable of precisely performing automatic and simultaneous reagent loading and aliquoting tasks and performing multistep assays with arbitrary sequences. The device is not intended to compete with other microfluidic technologies regarding ultra-low reaction volume. Instead, its freedom from tubing or pumping systems and easy operation makes it an ideal platform for routine high-throughput drug screening outside traditional microfluidic labs. The functionality and quantitative reliability of the 3D microfluidic device were demonstrated with a histone acetyltransferase-based drug-screening assay using the recombinant *Plasmodium falciparum* GCN5 enzyme, benchmarked with a traditional microtiter plate-based method. This arbitrarily accessible, multistep capable, low-cost, and easy-to-use device can be widely adopted in various combinatorial assays beyond high-throughput drug screening.

## 1. Introduction

High-throughput techniques are critically needed for efficient screening of pharmaceutically valuable lead compounds. The rapid progress of high-throughput screening (HTS) has enabled parallel analysis of thousands of reactions in order to identify effective compounds for a particular biological process. Most of the current HTS technologies involve robotics for automatic liquid and plate handling (96-, 384-, and 1536-well) [1,2,3,4,5]). Although the throughput has been increased tremendously by robotic technology as compared to manual operations [6], the high cost associated with the instrument has restricted many researchers from performing HTS independently. In addition, the volume required in the microtiter plate (100 μL and above) translates into a high cost for biological samples and drug libraries. As a result, there is a strong desire to develop low-cost technologies that require less sample and reagent consumption. To that end, the microfluidics-based drug-screening platform has been proposed due to its reduced reagent consumption, low cost, and high throughput [7,8,9,10].

In the past few years, significant progress has been achieved in developing microfluidics-based drug-screening components and systems. For example, the valve-based high-content microfluidic chamber arrays for a cell-based drug screening assay have been demonstrated [11,12,13,14,15]. The flow diffusive mixing-based systems provided a versatile method to generate on-chip concentration gradients [16,17,18,19,20,21]. The droplet-based microfluidics has shown nanoliter to picoliter compartmentalization in a high-throughput manner [22,23,24,25,26]. In addition, a low-cost slip chip provided a facile and economic method for performing microfluidic reactions without pumps or valves [10]. Recently, low-cost and easy-to-use 3D microfluidic chips have been introduced for high-throughput screening [27,28]. Despite impressive progress in microfluidics-based drug screening, challenges still remain. First, performing a multistep (>2 steps) combinatorial assay is a big challenge for most existing systems due to the difficulty in fluidic control. Second, microfluidic systems that require peripheral tubing and pumping system are too complex to operate by non-microfluidic researchers. Third, though there are commercial microwell plates in the volume range of a few microliters (e.g., Corning, Labcyte etc.), the reagent loading and aliquoting schemes are still labor-intensive due to its serial nature. As a result, there is a strong desire to develop an easy-to-use, low-cost, and multistep-assay-compatible microfluidic system that can be used by ordinary labs to perform HTS.

In this study, we describe an arbitrarily accessible, tubing-and-pumping-system-free, and multistep-assay-compatible 3D microfluidic device for HTS that can be routinely adopted by ordinary labs. Our microfluidic devices use a few hundred nanoliters to microliter to avoid the evaporation challenges common in nanoliter- to picoliter-scale multistep assays [29,30]. The device consists of sample-loading chips and auto-aliquoting chips for rapid, precise, automatic, and simultaneous heterogeneous/homogeneous reagent array formation. As a proof of concept, the device was validated with a recombinant *Plasmodium falciparum* GCN5 (*Pf*GCN5) enzyme-based drug-screening assay. The *Pf*GCN5 enzyme plays an important role in the regulation of chromatin structure and thus gene transcription in malaria parasites [31,32]; inhibition of *Pf*GCN5 activity is believed to be a potential target for novel chemotherapies of malaria [33,34,35]. The excellent quantitative agreement between the arbitrarily accessible 3D microfluidic device and the gold-standard 96-well microtiter plate demonstrates the reliability, robustness, and accuracy of the 3D microfluidic device. As the tubing-and-pumping-system-free 3D microfluidic device capable of performing multistep combinatorial assays with arbitrary sequences, we believe it offers unprecedented opportunities for low-cost and high-throughput drug screening in various laboratory settings. 

## 2. Materials and Methods

### 2.1. Materials and Chemicals

Polymethylmethacrylate (PMMA) sheets were from ePlastics. Food dye was from AmeriColor. HAT Activity Fluorometric Assay Kit was obtained from Biovision (Biovision Incorporated, Milpitas, CA, USA). Calcein and histone acetyltransferase (HAT) inhibitors (curcumin, cyclopentylidene-[4-(4′-chlorophenyl) thiazol-2-yl] hydrazine (CPTH-2), and (2R, 3S)-rel-4-methylene-5-oxo-2-propyltetrahydrofuran-3-carboxylic acid (MB-3)) were obtained from Sigma-Aldrich (St. Louis, MO, USA). 

### 2.2. Device Design and Fabrication 

The 3D microfluidic device consists of (1) a sample-loading chip; and (2) an auto-aliquoting chip (Figure S1). Both chip patterns were designed in AutoCAD 2015 (Autodesk Inc., San Rafael, CA, USA). The chips were fabricated with PMMA, which was patterned by the CO_2_ laser cutting machine (Epilog Laser Inc., Golden, CO, USA) with a power of 100%, a speed of 25% (for sample-loading chip of 1.6 mm thick), 30% (for auto-aliquoting chip of 1 mm thick), 60% (for spacer of 0.8 mm thick), and a frequency of 5000 Hz. After laser cutting, both chips were thoroughly cleaned in distilled water and isopropyl alcohol for 10 min. Both chips were then sandwiched between two metal plates and annealed in the oven for 24 h at 90 °C for stress relief. The sample-loading chip was then used as it is, whereas the auto-aliquoting chip was surface-coated with a thin layer of hydrophobic wax by laminating with the wax paper at 120 °C (Apache AL13P). The fabricated chips were kept in a sealed package before use. More detailed fabrication information can be found in Supplementary Materials.

### 2.3. Device Operation

**Figure 1:**
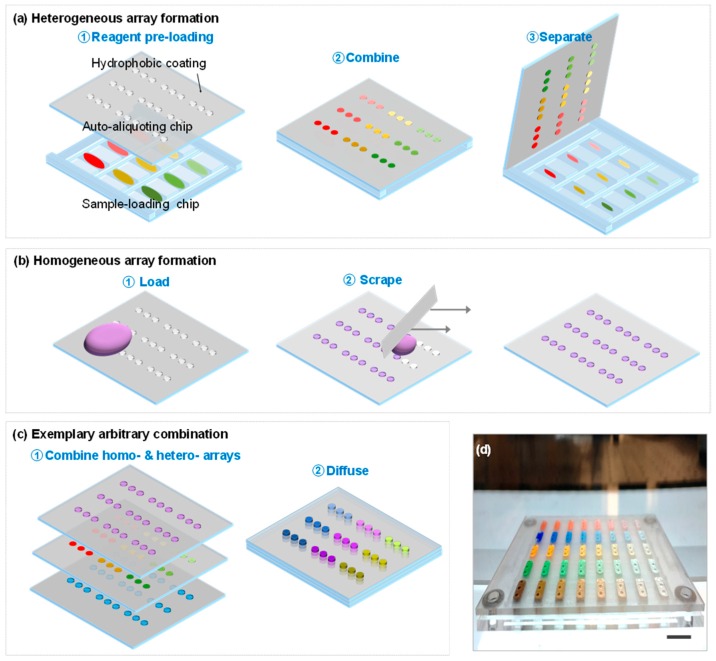
Schematic demonstration and photograph of the 3D microfluidic device. (**a**) Heterogeneous array formation. Heterogeneous reagents were loaded by high-throughput pipetting system onto the sample-loading chip. The pinning effect holds the liquid on each sample-loading island. By combining and separating the auto-aliquoting chip with the sample-loading chip (with a 0.8 mm high spacer), a heterogeneous reagent array is automatically formed; (**b**) Homogeneous array formation. Homogeneous reagent (e.g., enzyme solution or histone acetyltransferase (HAT) reaction mixture solution) was simply scraped over the wax-treated auto-aliquoting chip; (**c**) Exemplary arbitrary combination of three auto-aliquoting chips. The mixing of various reagents is achieved by the diffusion process; (**d**) Photograph of the device with the auto-aliquoting chip placed on top of the sample-loading chip. Note the schematic is not drawn to scale. The scale bar in the photograph is 1 cm.

**Figure 2:**
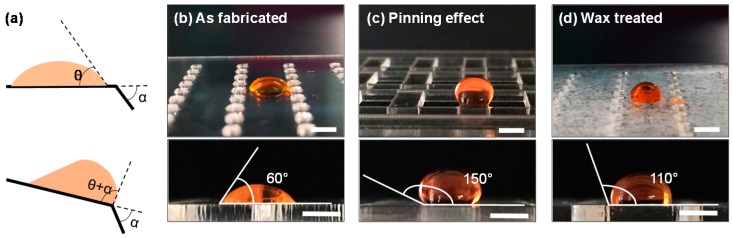
Pinning effect and chip surface treatment. (**a**) A droplet on a solid surface with a contact angle of θ, which will be increased up to θ + α when moving towards a three-phase edge; (**b**) The as-fabricated PMMA chip shows a contact angle of 60°; (**c**) The droplet pinned on the sample-loading chip shows a 150° contact angle; (**d**) The wax treated auto-aliquoting chip shows a contact angle of 110°. The scale bars in the images are 3 mm.

The arbitrarily accessible 3D microfluidic device starts in a high-throughput manner to form heterogeneous or homogeneous reagent arrays. (1) For heterogeneous array formation (Figure 1a), reagents with various concentrations were prepared and diluted in a 96-well plate and then dispensed onto the sample-loading chip with an HTS-compatible pipetting system (left illustration in Figure 1a). Afterward, the auto-aliquoting chip was simply placed on top of the sample-loading chip, with a spacer of 0.8 mm height (middle illustration in Figure 1a). The auto-aliquoting chip was then separated from the sample-loading chip. Due to the capillary force, the auto-aliquoting chip efficiently formed a uniform and heterogeneous reagent array, each of a volume of ~2 μL (right illustration in Figure 1a); (2) For homogeneous array formation (Figure 1b), a facile scraping process is sufficient to form a uniform array with the help of the capillary force. After forming various desired heterogeneous and homogeneous reagents arrays, a combinatorial assay can be performed by aligning and arbitrarily combining these microarrays (Figure 1c). Figure 1d shows the photograph of the device with the auto-aliquoting chip placed on top of the sample-loading chip. We did not find any liquid evaporation issues during the time course of the experiment.

### 2.4. Device Validation

To evaluate the uniformity and reaction performance of the 3D microfluidic device, we carried out validation experiments using the plain food dyes as well as the fluorescent calcein dye. For the food dye experiment, different colored food dyes were loaded and aliquoted to form a heterogeneous array and a homogenous array, using the methods mentioned above. For the fluorescent calcein dye experiment, calcein with concentrations ranging from 0.02 μM to 2.5 μM was used to form a heterogeneous array. A homogeneous array was formed with 1.25 μM calcein using the scraping method. The homogeneous and heterogeneous chips were then aligned and combined to evaluate the uniformity and accuracy of the device after mixing. 

### 2.5. PfGCN5 Enzyme Purification

To purify the *Pf*GCN5 HAT from the malaria parasite *Plasmodium falciparum*, a PTP (ProtC-TEV-ProtA) tag was added to the C-terminus of the endogenous *Pf*GCN5 gene in the malaria parasite as described before [36,37]. Briefly, ~1 kb fragment from the C-terminus end (without the stop codon) of *Pf*GCN5 was amplified and fused to the PTP tag and 3′ UTR region of the *P. berghei dhfr-ts* gene and cloned into the transfection vector pHD22Y with the human DHFR resistance cassette. Parasite transfection, drug selection, and cloning were performed as described [36,38]. Positive clones were verified by integration-specific PCR. The malaria parasites with PTP-tagged *Pf*GCN5 were cultured using a standard procedure [39] and the *Pf*GCN5 was purified by IgG beads under the native condition as described previously [36]. The purified protein was stored at −80 °C until enzymatic analysis.

### 2.6. HAT Assay for PfGCN5-Based Drug Screening

The 3D microfluidic device was applied in the HAT-based drug-screening assay to evaluate various drugs (inhibitors) against the purified *Pf*GCN5. In this assay, *Pf*GCN5 catalyzes the transfer of the acetyl group from acetyl–CoA to an H3 histone peptide, generating acetylated peptide and CoA–SH. The CoA–SH reacts with the developer to produce signaling fluorophore that is detected at Ex/Em = 535/587 nm. We first performed the reference experiment to investigate the compatibility of the 3D microfluidic device to the HAT-based assay and to establish the standard curve for the end-product (CoA–SH) according to the manual of HAT Activity Fluorometric Assay Kit. In addition, a pilot HAT-based drug-screening assay using positive controls (*Pf*GCN5 enzyme and HAT reaction mixture) and negative controls (buffer and HAT reaction mixture) was carried out on the 3D microfluidic device to determine the Z′-factor and thus the HTS potential of the assay [40]. Finally, several established inhibitors against the *Pf*GCN5 enzyme, including MB-3, CPTH-2, and curcumin [34,41,42], were used as reference drugs on the 3D microfluidic device and compared with the gold-standard 96-well microtiter plate method. The *Pf*GCN5 inhibitors were loaded onto the auto-aliquoting chip by the heterogeneous array formation process as described above, whereas *Pf*GCN5 and the HAT reaction mixture solution were loaded by the homogeneous array formation process. Detailed information of the experiment and the determination of the Z′-factor and the half maximal inhibitory concentration (IC_50_) are provided in the Supplementary Materials. The inhibitor chip, *Pf*GCN5 chip, and the HAT reaction mixture solution chip were then aligned and combined with an adaptor and incubated in a custom-built box at room temperature for 40 min in the dark. The combined chips were put on the homemade adapter and analyzed by the Typhoon scanner (GE 9410). With the adapter, the combined plates are 2 mm away from the surface of the scanner to prevent contaminating the scanner. We choose the 3 mm height mode for scanning with Ex/Em = 532/580 nm. The images were analyzed by ImageJ (NIH).

## 3. Results and Discussion

### 3.1. Device Design Theoretical Considerations

The sample-loading chip relies on the pinning effect to operate. For a liquid of an equilibrium contact angle θ on a solid surface moving towards a three-phase (liquid/vapor/solid) edge, the droplet will be pinned at the edge due to the increased liquid/vapor interface area and the increased activation barrier to move. The contact angle on the edge will increase from θ to θ + α and the droplet cannot move over the edge until the contact angle exceeds θ + α, as shown in Figure 2a, where α is the surface bending angle [43,44,45]. Our sample-loading chip was fabricated by cutting through rectangle-shaped windows with α = 90°, thus the maximum contact angle of a droplet on the sample-loading island is θ + 90°. As shown in Figure 2b,c, the as-fabricated PMMA chip has a contact angle of 60° and the measured contact angle on the edge of the sample-loading island is 150°, corresponding well to the theoretical prediction. This tremendously increased the contact angle and activation energy and helped prevent reagent leaking and mixing during the operation of the sample-loading chip. 

Another important aspect of the device operation is the surface wettability. For an as-fabricated PMMA chip without hydrophobic treatment, the observable liquid trace would be left on the chip surface. As a result, a hydrophobic surface is desirable. Accordingly, we performed wax treatment on the auto-aliquoting chip to obtain a hydrophobic surface. As shown in Figure 2d, the contact angle on the wax-treated auto-aliquoting chip increased to 110°. The wax on the device surface reduces the surface free energy and thus increases the liquid contact angle, consistent with the previous report [46]. The hydrophobic property of the auto-aliquoting chip made the subsequent operations of the 3D device more reliable. First, it minimized the liquid trace left on the chip surface during the array formation processes. Second, it prevented the liquid from leaking out when several auto-aliquoting chips were combined for reagent mixing and reaction. 

The heterogeneous array formation process is achieved by the capillary force. Once the auto-aliquoting chip is in contact with the sample-loading chip, the capillary force automatically drives the liquid into the auto-aliquoting chip array. At equilibrium, the upward force $F_{up}=2\pi a\gamma_{LV}\cos\theta$ is in balance with the downward gravity force $F_{down}=\rho gh\pi a^{2}$, where $\gamma_{LV}$ is the surface tension between liquid/vapor, a and h are the radius and the depth of the well, respectively, θ is the contact angle of liquid on the solid surface, ρ is the liquid density, and g is the acceleration of gravity [47]. For effective liquid loading, the capillary force needs to overcome the gravity force, which requires $a\leq 2\gamma_{LV}\cos\theta /\rho gh$. Each well in the auto-aliquoting chip used in the experiment has a radius of 750 μm and height of 1000 μm. This results in a capillary force of 171 μN, much larger than the gravity barrier of 17.3 μN, and thus an efficient sample auto-aliquoting process is guaranteed (Figure 1a). 

### 3.2. Quantitative Performances of the 3D Device

**Figure 3:**
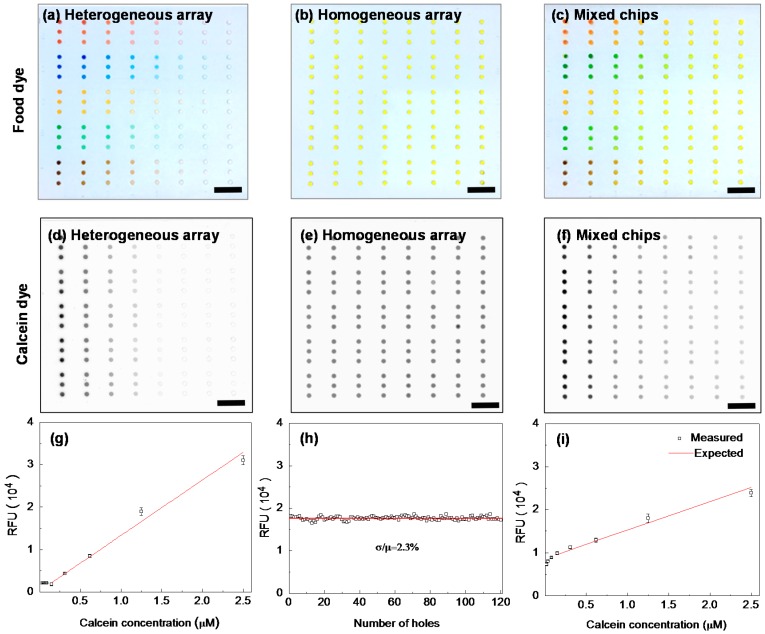
Validation of the 3D microfluidic device with plain food dyes and fluorescent calcein dye. (**a**–**c**) Food dye validation. The image shows the heterogeneous array chip consisting of different colors (**a**); homogeneous array chip of a single color (**b**); and the mixed chips (**c**); (**d**–**f**) Fluorescent calcein dye validation. The scanning images show the heterogeneous array chip (**d**); homogeneous array chip (**e**); and the mixed chips (**f**); (**g**–**i**) Measured fluorescence intensity for the heterogeneous array (**g**); homogeneous array (**h**); and the mixed chip (**i**); corresponding to images in (**d**–**f**); respectively. The fluorescence variation (σ/μ) within a homogeneous array is ~2.3% for 120 representative wells. The excellent agreement between the measured and the expected fluorescence intensity in the mixed chip shows that the mixing is reliable and thorough. Error bars correspond to 15 replicates. The scale bars in the images are 10 mm.

To establish the proof of concept for the proposed device, we tested the 3D microfluidic device with plain food dyes and a fluorescent calcein dye. We assessed the 3D microfluidic device by evaluating the loading efficiency, uniformity, and accuracy. Figure 3a–c shows the testing results with the food dyes. A heterogeneous array was generated with food dyes of different colors by a heterogeneous array formation process (Figure 3a). No observable dye trace was left over the auto-aliquoting chip surface after combining/separating with the sample-loading chip, indicating a highly efficient and effective array formation process. The homogeneous array with a single dye was formed by a homogeneous array formation process (Figure 3b). Due to the strong hydrophobicity of the chip surface, the scraping process only filled the wells without leaving a liquid trace on the chip surface. By combining these two arrays, a mixing process occurs through the diffusion process (Figure 3c). The color gradients indicated an effective mixing process. Leaking was not observed between the two arrays, indicating no cross-contamination during the combining process. The result with the food dyes validated the operating principle of the 3D microfluidic device.

To further characterize the quantitative performance of the 3D microfluidic device, we carried out the test with the fluorescent calcein dye. A heterogeneous array was formed with different concentrations of calcein dye (0.02, 0.04, 0.08, 0.16, 0.31, 0.63, 1.25, and 2.50 μM) using the heterogeneous array formation process, while a homogeneous array was formed with 1.25 μM of calcein using the homogeneous array formation process (Figure 3d,e). Again, no dye trace was detected on the array surfaces. Figure 3f shows the image of the mixed chips after combining the heterogeneous and homogeneous arrays. The quantitative performance can be evaluated from images of Figure 3d–f. First, Figure 3g shows the measurement of the fluorescence intensities of the heterogeneous array. The excellent fit (*R*^2^ = 0.98) in the linear range and small variations among replicates demonstrate that the heterogeneous array formation process is successful and reliable. Second, Figure 3h shows the measurement of the fluorescence intensities of the homogeneous array formed by scraping method. The fluorescence intensity variation of each array well was less than 2.3%, indicating a uniform homogeneous array formation process. Figure 3i shows the measured and the expected fluorescence intensity for the combined chip from Figure 3d,e. The excellent quantitative agreement confirms a thorough reaction mixing was achieved. 

### 3.3. PfGCN5-Based Malaria Drug Screening 

**Figure 4:**
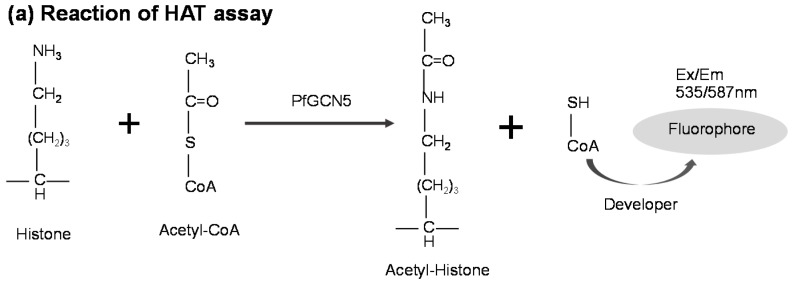

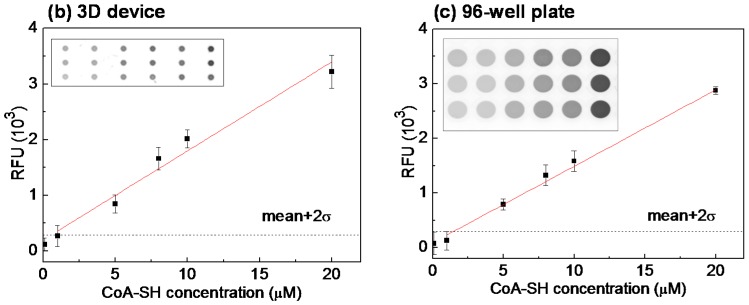
(**a**) Schematic illustration of HAT-based assay reaction. The *Pf*GCN5 enzyme catalyzes the acetylation and generates CoA–SH. The CoA–SH then reacts with the developer to produce signaling fluorophore; (**b**,**c**) the fluorescence intensity as a function of CoA–SH concentration on the 3D microfluidic device (**b**) and the 96-well microtiter plate (**c**). The insets show the scanning images corresponding to the plots in (**b**,**c**). The reaction volume in the 3D microfluidic device is 2 μL, 50 times less than the 100 μL in the 96-well microtiter plate. The red line is fitting for the linear range, which shows a good fit of *R*^2^ = 0.93 and 0.98 for the 3D microfluidic device and the 96-well microtiter plate, respectively. The dashed line indicated the optical scanner’s limit of detection, which is determined as two standard deviations above the background from the negative control (buffer and HAT reaction mixture). Error bars correspond to three replicates.

After validation of the device performance using dyes, we performed drug screening using the *Pf*GCN5 HAT on the 3D microfluidic device. We first performed a control experiment to investigate the suitability of the 3D microfluidic device for the HAT-based assay and to establish a standard curve for the end-product (CoA–SH) according to the manual of HAT Activity Fluorometric Assay Kit. The result was compared with that of the 96-well microtiter plate method as the gold standard. Figure 4a illustrates the reaction associated with the HAT-based assay [48]. The *Pf*GCN5 enzyme catalyzes the acetylation reaction that generates CoA–SH. The CoA–SH reacts with the developer to produce a signaling fluorophore. Figure 4b,c show the fluorescence intensity as a function of CoA–SH concentration reacting with the developer in the 3D microfluidic device and the 96-well microtiter plate, respectively. The plots show the standard curve of CoA–SH with a good fit for the 3D microfluidic device (*R*^2^ = 0.93) and the 96-well microtiter plate (*R*^2^ = 0.98), respectively. While the 3D microfluidic device consumes only 2 μL of reagents per reaction (a 50 times reduction as compared to the volume of 100 μL in a 96-well microtiter plate), the quantitative performance of the 3D microfluidic device and the 96-well microtiter plate remained the same (Figure 4b,c). This confirmed the compatibility of the 3D microfluidic device for the HAT-based drug-screening assay. 

**Figure 5:**
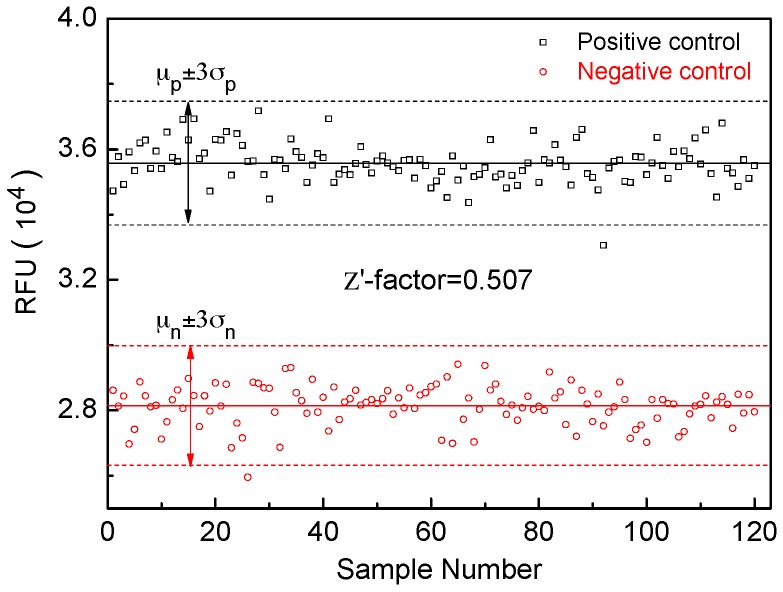
Z′-factor determination of the HAT-based drug-screening assay on the 3D microfluidic device. The solid horizontal lines show the mean values of the positive control and negative control. Black and red dash lines display 3 standard deviations (σ) from the mean value of positive control and negative control data set. The Z′-factor is determined to be 0.507 according to $Z\prime =1-(3\sigma_{p}+3\sigma_{n})/|\mu_{p}-\mu_{n}|$. A Z′-factor in the range between 0.5 and 1 indicates that the assay is suitable for HTS.

Pilot HAT-based drug-screening assays using positive and negative controls were carried out on the 3D microfluidic device to determine the Z′-factor, an indicator of the HTS potential of the assay [40]. Figure 5 shows the fluorescence intensity of the positive and negative controls of the HAT-based drug-screening assay. The positive controls have a mean value of 35,576 and a standard deviation of 613. The negative controls have a mean value of 28,149 and a standard deviation of 608. The Z′-factor determined from 120 replicates in three independent experiments on the 3D microfluidic device was 0.507, suggesting that it is suitable for HTS [40]. The small variation of positive controls and negative controls from different experiments also indicates the stability and reproducibility of the 3D microfluidic device. 

**Figure 6:**
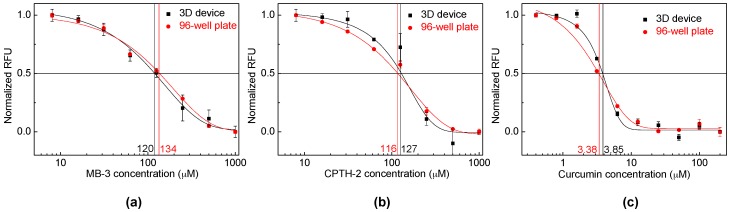
Drug response curves of the *Pf*GCN5 enzyme to (**a**) MB-3; (**b**) CPTH-2 and (**c**) curcumin on the 3D microfluidic device (**black**) and 96-well microtiter plate (**red**). *Pf*GCN5 was tested under serial concentrations of drugs. The plots show the normalized relative fluorescence unit as a function of drug concentration, where IC_50_ is the drug concentration at which normalized relative fluorescence units (RFU) is 0.5, indicating 50% inhibition. The extracted IC_50_ are indicated on the plot. Error bars correspond to three replicates.

After confirming the compatibility of the HAT-based assay with the 3D microfluidic device, MB-3, CPTH-2, and curcumin were used as reference compounds on the 3D microfluidic device and compared with the 96-well microtiter plate method. Figure 6 showed that MB-3, CPTH-2, and curcumin are strong inhibitors of the *Pf*GCN5 HAT, consistent with previous reports [34,41,42]. Higher concentrations of these compounds inhibited more *Pf*GCN5 enzyme and resulted in less CoA–SH (Figure 4a), leading to lower fluorescence intensity. The IC_50_ values determined on the 3D microfluidic device and in the 96-well microtiter plate were comparable, with a variation smaller than 11% (120 and 134 μM for MB-3, 127 and 116 μM for CPTH-2, 3.85 and 3.38 μM for curcumin using the 3D microfluidic device and the 96-well microtiter plate, respectively). The excellent agreement between the two results demonstrates the reliability, robustness, and accuracy of the 3D microfluidic device.

### 3.4. Versatility and Adaptability of the 3D Microfluidic Device

It is worth noting that we do not envision the 3D microfluidic device to compete with picoliter to nanoliter technologies (e.g., droplet microfluidics [22,25]). The advantages of our 3D microfluidic device are the following: (1) High-throughput. By adopting the heterogeneous and homogeneous array formation processes, the 3D microfluidic device allows parallel reagent loading and aliquoting in a much faster method (a few seconds) than the serial loading process using a pipetting system (minutes to hours); (2) Multiplexity. With the automatic heterogeneous array formation process from a library of reagents, various reagents can be simultaneously arrayed using the 3D microfluidic device. It is noteworthy that reagents from different auto-aliquoting chips can be mixed in parallel by the combinatorial process, ensuring simultaneous incubation for each individual reactions; (3) Versatility. The combinatorial nature of the 3D microfluidic device allows for any multistep assay with arbitrary incubation sequence. A device capable of performing the multistep assay is highly desirable since a variety of biological assays require a series of combinatorial reactions. Besides, the 3D microfluidic device is versatile with various readout methods, as the detection can be easily extended to various imaging setups; (4) Low-Cost. Both the device materials (PMMA, 0.04 dollars per chip) and the fabrication process (laser cutting) is cost-effective. The device operation requires no tubing and pumping instruments and thus can be easily adapted to other labs without microfluidics experience and microfluidics infrastructures.

## 4. Conclusions

We report an easy-to-use, scalable, and cost-effective 3D arbitrary microfluidic device for an HTS purpose. Using an HAT-based malaria drug-screening assay as a model system, we evaluated the drug-screening performance and benchmarked it with the gold-standard 96-well microtiter plate method. The demonstrated 3D arbitrary microfluidic device is capable of easily performing automatic and simultaneous reagent aliquoting due to its parallel nature, as well as performing an arbitrary sequence of assay steps. The device is not intended to compete with other microfluidic technologies regarding ultra-low reaction volumes (e.g., pL–nL in droplet microfluidics [22,25]). Instead, the operation of the device does not require any peripheral tubing or pumping systems, which makes it an ideal platform for ordinary labs to perform high-throughout, multistep drug-screening assays. The current device can be easily scaled up to an array size of 1056 with reagent consumption of ~100 nL per reaction. A thorough screening of a small chemical library against *Pf*GCN5 is currently underway. We believe that the 3D arbitrary microfluidic device is not only valuable as a general platform for high-throughput drug-screening, but could also be widely extended to various other bioassays.

## Supplementary Materials

The supplementary materials are available online at http://www.mdpi.com/1424-8220/16/10/1616/s1. Supplementary text includes the design and fabrication of the 3D microfluidic device and the experiment operation of the HAT-based assay. Figure S1 shows the design schematic and images of (a) the sample-loading chip and (b) auto-aliquoting chip. Figure S2 shows the PfGCN5 activity in HAT assay.
