# Supplementary material for: Arbitrarily Accessible 3D Microfluidic Device for Combinatorial High-Throughput Drug Screening
Source: Sensors (Basel). 2016 Sep 29;16(10):1616. doi: 10.3390/s16101616 (PMC5087404; doi:10.3390/s16101616)
Supplement: Supplementary file 1 [file sensors-16-01616-s001.pdf]

Supplementary Materials: Arbitrarily Accessible 3D Microfluidic Device for Combinatorial High-Throughput Drug Screening

Zhuofa Chen, Weizhi Li, Gihoon Choi, Xiaonan Yang, Jun Miao, Liwang Cui and Weihua Guan

Design and Fabrication of the 3D Microfluidic Device

The sample-loading chip and the auto-aliquoting chip were designed by AutoCAD 2015 (Autodesk Inc., San Rafael, CA, USA) and transferred to the Epilog laser engraving and cutting system (Epilog laser Inc., Golden, CO, USA). The PMMA sheet was fabricated by the CO_2 laser cutting machine with a power of 100%, a speed of 25% (for sample-loading chip of 1.6 mm thick), 30% (for auto-aliquoting chip of 1 mm thick), 60% (for spacer of 0.8 mm thick), and a frequency of 5000 Hz. The devices were sandwiched with metal plates and annealed in the oven for 24 h at 90 °C for stress relief. The sample-loading islands on the sample-loading chip were built by cutting discrete rectangle-shaped pinning windows. The wells on the auto-aliquoting chip were built by cutting through-holes of 1.5 mm in diameter. The sample-loading chip was 92 mm long, 72 mm wide, and 1 mm thick (Supplementary Materials Figure S1). Each sample-loading island on the sample-loading chip is corresponding to 3 wells (triplicates) on the auto-aliquoting chip. Though the pitch size of the 3D device can be reduced arbitrarily, the current design has a pitch size of 9 mm, compatible with the multi-channel pipette system. By optimizing the design, we can scale up the device to the 1056-well microarray platform (with 100 nL/well).

Experiment Operation of the HAT-Based Assay

(i) Standard curve of CoA–SH

We first performed the reference experiment to investigate the suitability of 3D microfluidic device to the HAT-based assay and to establish the standard curve for the end-product (CoA–SH). To validate the result from the 3D microfluidic device, we performed control experiment on the 96-well microtiter plate. In 96-well microtiter plate-based experiment, 20 μM CoA standard was diluted in the HAT assay buffer and made a discrete CoA standard concentration of 20 μM, 10 μM, 8 μM, 5 μM, 1 μM, 0.1μM, and 0 μM (as negative control). Added in 100 μL of each CoA standard solution into each well. Then 100 μL HAT reaction mixture was added into each well, which contents 64 μL buffer, 8 μL Histone3, 20 μL substrate mix, 4 μL developer, and 4 μL pico probe. The 96-well microtiter plate was then incubated at room temperature for 40 min in the dark. While in the 3D microfluidic device, CoA solution with various concentrations were pipetted onto the sample-loading chip using multichannel pipette (40 μL each). Combined the auto-aliquoting chip with the sample-loading chip, then separated the auto-aliquoting chip to form heterogeneous CoA solution array. The HAT reaction mixture was pipetted on another auto-aliquoting chip and scraped using a glass slide. At last, combined the two auto-aliquoting chips and incubated for 40 min at room temperature in the dark.

(ii) *Pf*GCN5 enzyme activity

After confirming HAT assay compatibility with the 3D microfluidic device, we studied the activity of the *Pf*GCN5 of the HAT assay in the 96-well microtiter plate and optimized the scanning time for the HAT-based drug-screening assay. Each 100 μL HAT reaction mixture contains 60 μL buffer, 8 μL Histone3, 20 μL substrate mix, 4 μL developer, 4 μL acetyl-CoA, and 4 μL pico probe. The *Pf*GCN5 (7.4 μg/mL) solution was prepared by diluting 10 μL *Pf*GCN5 in each 100 μL HAT assay buffer. Then added 100 μL HAT reaction mixture and 100 μL *Pf*GCN5 solution into each well. Supplementary Materials Figure S2 shows the fluorescence intensity versus to scanning time at 40 min, 50 min, and 60 min after mixing reagents. The result shows the excellent catalyzation of *Pf*GCN5 for

the histone acetylation process and the purified *Pf*GCN5 enzyme can be used for the following HAT-based drug-screening assay.

(iii) HAT-based drug-screening assay

After confirming the HAT assay compatibility with the 3D microfluidic device and the *Pf*GCN5 activity of HAT-based assay, several established inhibitors against *Pf*GCN5 enzyme, including MB-3, CPTH-2, and curcumin [1–3], were used as model drugs on the 3D microfluidic device and benchmarked with the standard 96-well microtiter plate. The curcumin, MB-3, and CPTH-2, were dissolved in sterile dimethyl sulfoxide (DMSO) and stored at −20 °C. These inhibitors were diluted in the HAT assay buffer to specific concentration immediately prior to use. In the 96-well microtiter plate, each 100 μL HAT reaction mixture contains 60 μL buffer, 8 μL Histone3, 20 μL substrate mix, 4 μL developer, 4 μL acetyl-CoA, and 4 μL pico probe. The PfGCN5 solution was prepared by diluting 10 μL PfGCN5 in each 100 μL HAT assay buffer. The MB-3, CPTH-2, and Curcumin was first diluted in the HAT assay buffer to 1 mM, 1 mM, and 100 μM, respectively and followed by doubling dilution. Each well contains 100 μL HAT reaction mixture, 100 μL drug, and 100 μL *Pf*GCN5. In the 3D microfluidic device, drugs with various concentrations were pipetted onto the sample-loading chip using multichannel pipette (40 μL each). Combined the auto-aliquoting chip with the sample-loading chip and then separated the auto-aliquoting chip and the heterogeneous drugs solution array was generated. The *Pf*GCN5 was pipetted on the auto-aliquoting chip and scraped using a glass slide. The HAT reaction mixture was loaded in the same way as the *Pf*GCN5. At last, combined the three auto-aliquoting chips and incubated for 40 min at room temperature in the dark.

Z′-Factor Analysis

The *Pf*GCN5 and the HAT reaction mixture were prepared as described above. We first loaded the HAT reaction mixture on one auto-aliquoting chip by scraping method. For the second auto-aliquoting chip, we scraped buffer on half of the chip (as negative control) and scraped *Pf*GCN5 solution on the other half of the auto-aliquoting chip (as positive control). At last, combined the two auto-aliquoting chips and scanned by the typhoon scanner. We repeated this experiment for three times and collected the representative data for Z′-factor calculation. The Z′-factor was determined to be 0.507 according to $Z' = 1 - (3\sigma_p + 3\sigma_n)/|\mu_p - \mu_n|$, where σ_p and μ_p is the standard deviation and mean value of the positive control, σ_n and μ_n is the standard deviation and mean value of the negative control [4].

IC$_{50}$ Fitting Model

The half maximal inhibitory concentration (IC$_{50}$) was calculated by fitting the data using the 4-parameter logistic model [5]:

$$Y = \frac{a - d}{1 + (X / c)^b} + d$$

where Y is the fluorescence intensity and X is drug concentration. a is the lower plateau (minimal value of the curve corresponding to highest drug concentration). d is the upper plateau (maximum value of the curve corresponding to the negative control without any drug). c is the concentration of drug corresponding to the fluorescence intensity midway between a and d. b is the slope factor of the curve.

Supplementary Figure

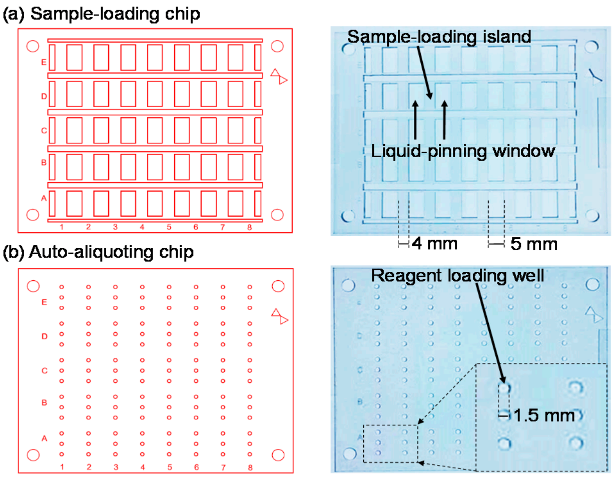

Figure S1. The design schematic and images of (**a**) sample-loading chip and (**b**) auto-aliquoting chip. The sample-holding island has a dimension of 4 mm by 11 mm, the pinning window between the sample-holding islands has a dimension of 5 mm by 9 mm. A zoomed-in photo shows the representative array of wells on the sample-loading chip, the wells have uniform diameter of 1.5 mm.

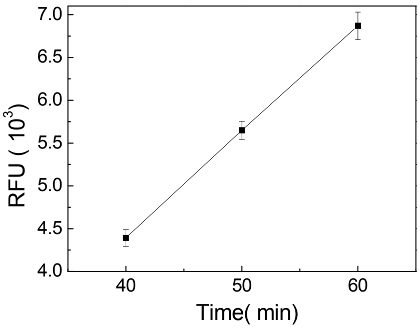

Figure S2. *Pf*GCN5 activity in HAT assay. Fluorescence intensity versus to scanning time at 40 min, 50 min, and 60 min after mixing.

Reference

1. Secci, D.; Carradori, S.; Bizzarri, B.; Bolasco, A.; Ballario, P.; Patramani, Z.; Fragapane, P.; Vernarecci, S.; Canzonetta, C.; Filetici, P. Synthesis of a novel series of thiazole-based histone acetyltransferase inhibitors *Bioorgan. Med. Chem.* **2014**, *22*, 1680–1689.
2. Shukla, A.; Singh, A.; Singh, A.; Pathak, L.P.; Shrivastava, N.; Tripathi, P.K.; Singh, M.P.; Singh, K. Inhibition of P falciparum pfATP6 by curcumin and its derivatives: A bioinformatic Study. *Cell Mol. Biol.* **2012**, *58*, 182–186.
3. Chimenti, F.; Bizzarri, B.; Maccioni, E.; Secci, D.; Bolasco, A.; Chimenti, P.; Fioravanti, R.; Granese, A.; Carradori, S.; Tosi, F.; et al Synthesis and biological evaluation of novel conjugated coumarin-thiazole systems. *J. Med. Chem.* **2009**, *52*, 530–536.
4. Zhang, J.H.; Chung, T.D.Y.; Oldenburg, K.R. A Simple Statistical Parameter for Use in Evaluation and Validation of High Throughput Screening Assays. *J. Biomol. Screen.* **1999**, *4*, 67–73.
5. Delean, A.; Munson, P.J.; Rodbard, D. Simultaneous analysis of families of sigmoidal curves: application to bioassay, radioligand assay, and physiological dose-response curves. *Am. J. Physiol.* **1978**, *235*, E97–E102.
